# Silver, bighead, and common carp orient to acoustic particle motion when avoiding a complex sound

**DOI:** 10.1371/journal.pone.0180110

**Published:** 2017-06-27

**Authors:** Daniel P. Zielinski, Peter W. Sorensen

**Affiliations:** Department of Fisheries, Wildlife, and Conservation Biology, University of Minnesota, St. Paul, Minnesota, United States of America; University of Auckland, NEW ZEALAND

## Abstract

Behavioral responses of silver carp (*Hypopthalmichthys molitrix*), bighead carp (*H*. *nobilis*), and common carp (*Cyprinus carpio*) to a complex, broadband sound were tested in the absence of visual cues to determine whether these species are negatively phonotaxic and the roles that sound pressure and particle motion might play mediating this response. In a dark featureless square enclosure, groups of 3 fish were tracked and the distance of each fish from speakers and their swimming trajectories relative to sound pressure and particle acceleration were analyzed before, and then while an outboard motor sound was played. All three species exhibited negative phonotaxis during the first two exposures after which they ceased responding. The median percent time fish spent near the active speaker for the first two trials decreased from 7.0% to 1.3% for silver carp, 7.9% to 1.1% for bighead carp, and 9.5% to 3% for common carp. Notably, when close to the active speaker fish swam away from the source and maintained a nearly perfect 0° orientation to the axes of particle acceleration. Fish did not enter sound fields greater than 140 dB (ref. 1 μPa). These results demonstrate that carp avoid complex sounds in darkness and while initial responses may be informed by sound pressure, sustained oriented avoidance behavior is likely mediated by particle motion. This understanding of how invasive carp use particle motion to guide avoidance could be used to design new acoustic deterrents to divert them in dark, turbid river waters.

## Introduction

Acoustic energy propagates through water as a traveling pressure wave with accompanying particle motion and is used by fish to mediate numerous life cycle functions including migration, communication, prey detection, and avoidance. To use sound efficiently, fish need to be able to both distinguish signals above background noise and then use this information to orient, or move in a directed fashion. While sound pressure, a scalar quantity, cannot provide fish with any immediate directional information on its own, particle motion, a vector quantity, is inherently directional and could. However, although the capacity for directional hearing in fish is relatively well described [1–4], only a handful of experimental studies have tested how it is mediated. These studies have shown that both sound pressure and particle motion can play very different, and independent roles in the oriented movement of fish seeking a sound source (positive phonotaxis) [5–9]. In contrast, although sound induced repulsion (negative phonotaxis) has also been described in a few species of fish, the sensory cues responsible for these responses have not yet been explicitly described so are unknown [10–14]. How fish might orient toward and away from sound sources has both basic implications for understanding how fish might use sound to meet their ecological needs as well as strong implications for how sound might be used to either attract or repel fishes of concern in the natural world. The present study characterized the orientation mechanisms used by three species of invasive carp as they avoided a sound source and the two sensory fields it created in the absence of visual cues.

All teleost fishes are similarly equipped to detect particle motion, but their abilities to detect sound pressure vary greatly. Particle motion is detected via a fish’s inner ear otolithic end organs, which function as accelerometers when their dense otoliths move in response to the acoustic field over a sensory epithelia with polarized hair cells [15]. Particle motion detection by its very nature has a distinct directional component. Conversely, sound pressure, which lacks directional information, is only detected with notable sensitivity by fish which possess an acoustic coupling (i.e. Weberian apparatus) between a gas-filled pocket (generally the swim bladder) and their inner ear [16–18]. Fish that have evolved notable sensitivity to sound pressure have a wider hearing bandwidth and greater sensitivity than species without specializations [4]. While both particle motion and sound pressure are also detected by the mechanosensory lateral line in all fishes, it only detects low frequencies (< 300 Hz) and only in the acoustic near-field [19–20], so sound sensitivity in fish is in most instances attributable to the inner ear.

Both sound pressure and particle motion based orientation mechanisms have now been described in two fish species by carefully describing the approach pathways they take to locate sound sources. In the first example, the female plainfin midshipman, *Porichthys notatus*, was found to locate the sound of calling mates in a featureless dim environment by swimming in a direction that had a near constant angle to the axis of acoustic particle motion [7–9]. In contrast, blinded mottled sculpin, *Cottus bairdi*, was found to use sound pressure to locate a dipole sound source (50 Hz) by swimming in a distinct zig-zag swimming pattern [5,6]. By zig-zagging, sculpin were seemingly able to assess the relative intensity of sound pressure at different locations, and thus orient. These two orientation strategies which employ particle motion and sound pressure are markedly different from each other. Orientation mechanisms have not yet been explicitly described for fish avoiding sound which is complicated because acoustic signal intensity drops with distance, making comparisons of relative intensity more difficult.

Two species of bigheaded carp from Asia, the silver carp (*Hypopthalmichthys molotrix*) and the bighead carp (*H*. *nobilis*) were introduced to the United States in the 1970s, and have become highly abundant and invasive in the Mississippi River. Because these fish adversely impact aquatic food webs [21–24] and one jumps; there is strong interest in developing technologies to block their expansion up the Mississippi River [25]. Similarly, the common carp (*Cyprinus carpio*), a related cyprinid from Eurasia [26], is also invasive and has been responsible for degrading millions of acres of shallow wetland ecosystems across the globe so there is interest in stopping its movement between waterways [27]. All carps are Ostariophysians and have well developed hearing abilities that include a heightened sensitivity to sound pressure, which is superior to that of many native North American fishes [28–30]. Accordingly, it has been proposed that acoustic deterrents might be used to block the access of invasive carps to critical habitat [10,11, 31–38]. Recently, Vetter et al. [10,11] demonstrated that large groups of silver carp and bighead carp exhibited negative phonotaxis to a complex outboard motor sound when tested in a well-lit arena with exposed speakers when sound was repeatedly played when fish approached a specific location. While the distance of the apparent centroid of the fish school was measured relative to the sound source, the positions and orientations of individuals were not tracked to determine specific angles of orientation to any sound cues, so whether the observed responses were oriented to the sound field, or influenced by the physical presence of the speaker (which was visible to fish) were unclear. The particle motion component of sound that was played and its possible role relative to carp bearing was also not assessed so its role was similarly unclear. Thus, while intriguing, the implications of this work to understanding orientation and deterrence to sound and its possible applications to riverine acoustic barriers in dark or turbid / featureless waters are unclear. Indeed, no study that we know of has determined the orientation mechanisms used by any fish to avoid sounds by precisely mapping movement relative to known sound pressure and particle motion fields in the absence of visual cues.

The present study investigated the nature of behavioral responses of silver, bighead, and common carp to a stationary, monopole sound source to characterize whether and how these species avoid complex sound in the absence of visual cues. Specific goals were to: (1) determine whether silver, bighead, and common carp are all negatively phonotaxic (i.e. move away from the sound source) to complex, broadband sounds in the absence of visual cues, and (2) test the relative roles of sound pressure and acoustic particle motion in this response. A complex, broadband outboard motor sound was used because it had already been tested by Vetter et al. [10,11] and had also previously been shown to induce physiological stress responses in the common carp [39].

## Materials and methods

### Experimental animals

Juvenile silver carp [mass: 120 ± 41 g (mean ± SD); total length: 237 ± 35 mm] and bighead carp [mass: 32 ± 16 g; total length: 139 ± 21 mm] were obtained from the Columbia Environmental Research Center (U.S. Geological Survey, Columbia, MO, USA) and held in circular 100-L tanks until needed. Bigheaded carp were fed a planktonic diet consisting primarily of Spirulina and Chlorella algae (see [40]) once a day between 10:00 h and 14:00 h. Common carp [mass: 416 ± 113 g; total length: 298 ± 25 mm] were caught in Casey Lake, MN, USA (45°01’22” N, 93°00’49” W) by pulsed DC electrofishing in July 2012 and transported to the laboratory, where they were maintained in tanks (1.5 m diameter x 50 cm deep). Common carp were fed pellets (Silver Cup, Utah) once a day between 10:00 h and 14:00 h. Fish were held at a 16h:8h (L:D) photoperiod and all holding and experimental tanks were supplied continuously with flow-through 20°C well water. All experimental procedures were approved by the University of Minnesota Institutional Animal Care and Use Committee (Protocol: 1201A08922), and all necessary federal and state permits for shipping and holding prohibited species were also obtained.

### Experimental setup

**Fig 1:**
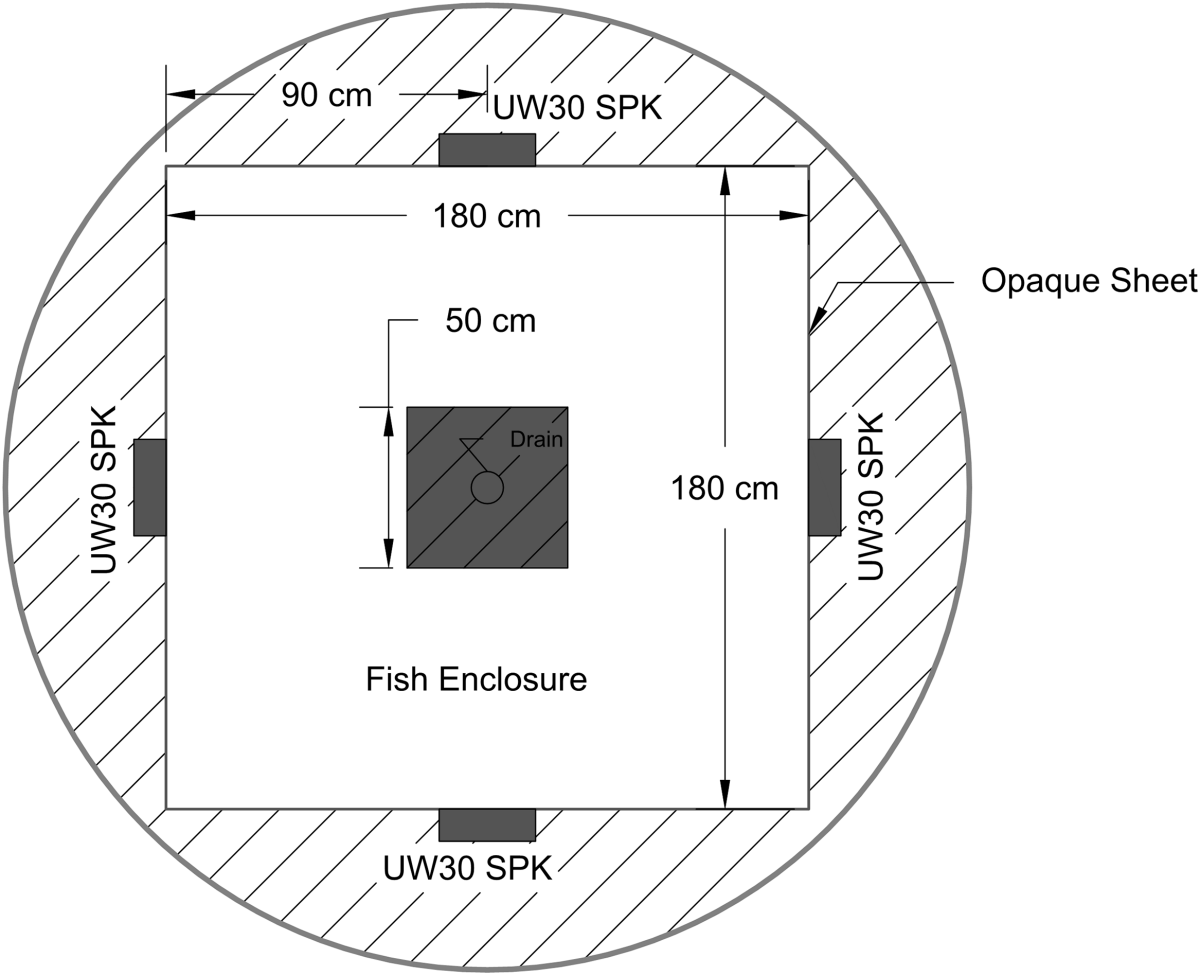
Schematic of the experimental tank showing the locations of the speakers, plastic enclosed testing arena, and drain cover box. The outside diameter of the tank was 3 m and the water depth was 30 cm. The tank was darkened by a black plastic tarp which covered it and illuminated by infrared lights.

Experiments were performed in a cylindrical fiberglass tank (3 m diameter, 2 m in depth) into which an internal square opaque plastic enclosure (1.8 m on a side, 50 cm high, 150 μm thick) had been placed to render the testing arena featureless (Fig 1). The center of the arena had a drain pipe which was also shielded with a black plastic box (50-cm high on each side). This tank was supplied with well water to a depth of 30 cm and aerated by airstones positioned outside of the enclosure in each corner. A black plastic tarp covered the entire tank and three infrared floodlights (840 nm) illuminated the inside of a darkened arena. Light levels were extremely low (0.5 μW/cm^2^) and the tank devoid of obvious visual cues, so it is highly unlikely that vision would have been useful to fish even though the common carp’s visual sensitivity extends into the infrared (870 nm) [41] (the visual sensitivity of bighead and silver carp has not been reported). Four UW30 speakers (output level 153 dB (ref. 1 μPa) at 1 m, frequency response 0.1 to 10 kHz, Electrovoice, MN, USA) were positioned outside the plastic arena (so they were not visible) at the center of each side using cables that acoustically separated them from the tank and set the center of the speaker 15-cm above the tank floor. A closed circuit video camera (Interlogix, NC, USA) was mounted 3 m above the tank bottom, and recorded at 30 frames per second through each experiment. Video files were later downloaded from a DVR and a custom Matlab (Mathworks, MA, USA) script was used for frame-by-frame analysis.

### Acoustic stimuli

**Fig 2:**
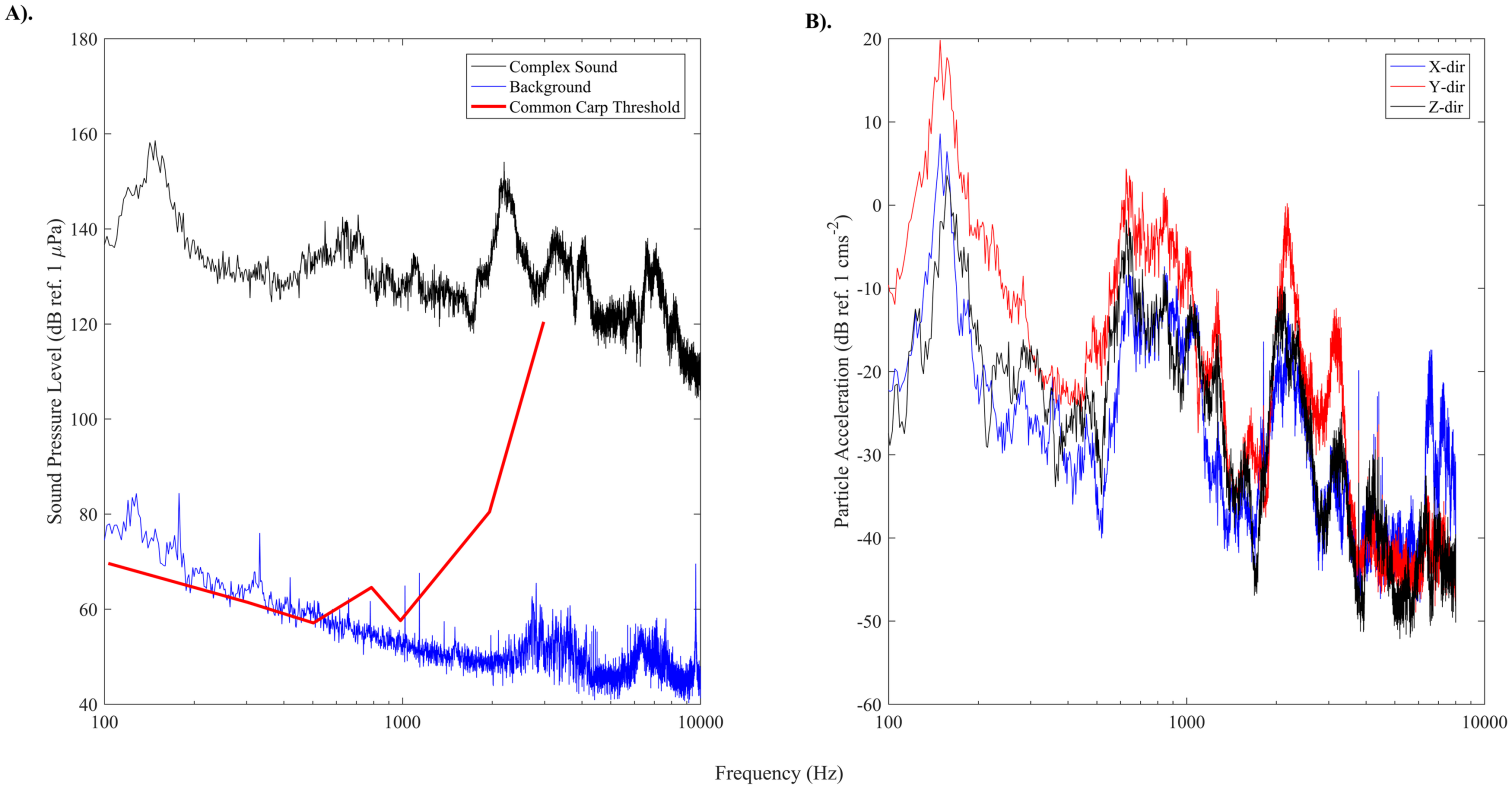
A) Sound pressure level power spectrum of the background noise, playback signal 5 cm from the speaker, and hearing threshold of common carp (from Popper, 1972). B) Particle acceleration measurement in decibels (ref. 1 cms^-2^) in each direction at a point 5 cm in front of the speaker. Sound pressure level and particle acceleration measurements are provided at 1 Hz bandwidth. Note, the 1 cms^-2^ limit suggested by Knudsen et al. [42] is at 0 dB (ref. 1 cms^-2^).

**Fig 3:**
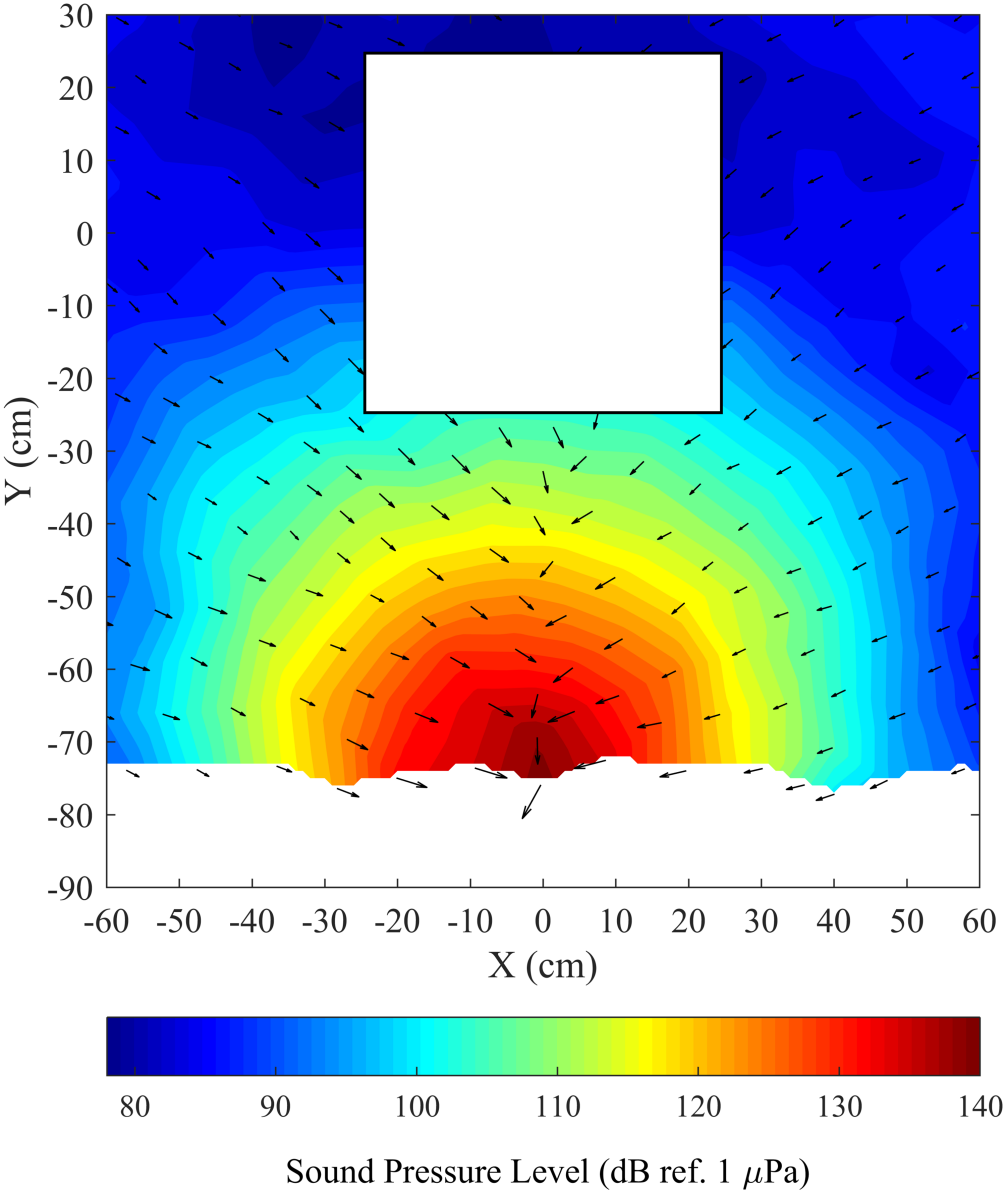
Plan view of sound pressure level in dB (ref 1 μPa) at 2000 Hz in the enclosure at a depth of 9 cm from the tank bottom with particle acceleration vectors in *xy*-plane. Particle acceleration magnitude is calculated using only the acceleration in the *x*- and *y*-directions. The speaker was hidden behind a plastic enclosure and located at 0 cm on the X-axis, with the center of the projector face 15 cm from the tank bottom. Contours do not extend to 90 cm because acoustic instrumentation could not be placed closer to the plastic enclosure.

We tested a complex, broadband sound that had been recorded from a 40 hp outboard motor. Sound was played for 120-s via speakers when fish were within 30 cm (see experimental design section for details) and produced a peak sound pressure level (SPL) of approximately 150 dB (ref. 1 μPa) directly in front of each speaker with most of its energy within two peaks around 150 Hz and 2000 Hz (Fig 2A). Similar to Vetter et al. [11], the sound field measured in the tank differed slightly from the original signal at low frequencies due to the speaker frequency response (S1 Fig) The frequency range of the playback signal overlapped the hearing range for common carp [28] which is also very similar to the hearing ranges of silver and bighead carp [29,30], 50–3000 Hz (Fig 2A). The background sound pressure level was below 80 dB (ref. 1 μPa) throughout the enclosure when inflow and airstones were turned off. Sound pressure contours decreased in a radial fashion away from the speaker (Fig 3) and differed by less than 5% between all four speakers (see S2 and S3 Figs for sound pressure contour of entire enclosure and radial attenuation of sound pressure level). Particle acceleration was approximately 20 dB (ref. 1 cms^-2^) in front of the speaker with most of the energy within three peaks around 150, 1000, and 2000 Hz (Fig 2B). Particle acceleration vectors in the *xy*-plane were orthogonal to sound pressure contours, pointing towards (or away from) the projector (Fig 3). Particle acceleration was similar in all three directions throughout the tank (S4 Fig).

Both sound pressure levels and particle acceleration were mapped on a Cartesian grid throughout the tank at 5-cm intervals within 30-cm of each of the four speakers and 15 cm above the tank bottom. Sound measurements were made using a PVC probe similar to that used by Zeddies et al. [8], which contained a C55 hydrophone (usable frequency range of 0.008–100 kHz and a sensitivity of approximately -163.5 dB ref 1V/μPa, Cetacean Research, WA, USA) and a PCB model W356A12 triaxial accelerometer (usable frequency of 0.5–5000 Hz and sensitivity of approximately 100 mV/ (m/s^2^), PCB Piezoelectronics, NY, USA). The sound pressure signal was sampled at 44.1 kHz and fed through a TASCAM US-122mkII (TASCAM, CA, USA) audio interface, digitized, and stored on a windows-based computer. The accelerometer was neutrally buoyant because it had been embedded in an extruded polystyrene foam enclosure. The acoustic particle acceleration signal was conditioned using a PCB 482C05 conditioner and fed through a USB-1208FS-Plus data acquisition board (Measurement Computing, MA, USA) sampling each channel at 16 kHz. At each location, a 10 s sample was split into 10 signal ensembles and averaged. Data acquisition hardware was controlled by a custom graphical user interface operating in Matlab, which was also used to analyze and transform the pressure and particle acceleration waveforms into the frequency domain.

### Experimental design

Tests were conducted between 10:00 and 16:00 h between December 2013 and August 2014. Fish were tested as groups of three individuals of the same species to facilitate natural shoaling behavior and reduce stress [43,44]. Prior to testing, fish were allowed to acclimate, shoal, and move freely. Acclimation times differed by species and had been determined beforehand by extensive pilot tests as the periods of time required by fish to start to explore tanks and feed when offered food (130 min for common carp, 20 min for silver carp, and 24 h for bighead carp). Water inflow and airstones were turned off 10 min prior to the start of each trial. After the 150 s pre-test period (control), the test sound was played once two individual carp swam within 30-cm of any one of the four speakers, at which time that speaker was turned on for 150-s (treatment). The 30-cm distance was used as a threshold because sound mapping showed it to coincide with both the region of maximum sound pressure and the 1 cms^-2^ particle acceleration limit for avoidance behaviors previously prescribed by Knudsen et al. [42] and Karlsen et al. [45]. This procedure was repeated for four trials (each with a control and treatment period) until all four speakers had been used once (time between trials varied, and fish could not learn order of testing). After testing, fish were removed and placed into a control tank. Each species was tested 7 times and no fish were reused.

### Analysis of fish distribution and orientation

Data were evaluated in two steps. Step one evaluated fish distribution (i.e., avoidance) while step two determined the tracks that individual carp followed (i.e., orientation) and then evaluated how fish oriented to known sound fields to discern the orientation mechanisms they were using.

#### Fish distribution and avoidance

For the first analysis, the percent time each fish spent within 30-cm of the active speaker (or the soon-to-be active speaker for control periods) was calculated after viewing videos. This was accomplished by recording the *x* and *y* coordinates of each fish’s head within each group of three at 5 s intervals (i.e. once every 150 frames). Initial plots of fish movement showed that fish rapidly moved in the first few seconds of sound exposure before assuming a more constant distribution (S5 Fig), so we chose to exclude the first 30 s of their behavior from this particular analysis to assess their long-term responses and avoidance. For each group of fish (and trial), the percent time fish spent within 30-cm of the active speaker (after the first 30 s) was calculated by dividing the total number of times any fish was within 30-cm of the active speaker by the total number of data points. These values were examined for normalcy (Shapiro-Wilk tests) and appropriate paired comparisons performed. Because the data were not normally distributed nonparametric Mann-Whitney U-tests [46] were used to compare differences in the percent time that groups of fish of each species spent within 30-cm of an active speaker between matched control and treatment periods (i.e. Control #1 vs. SPK#1, Control #2 vs. SPK#2, etc.). Significance was determined at P<0.05. All assumptions of these tests were met.

#### Fish orientation

**Fig 4:**
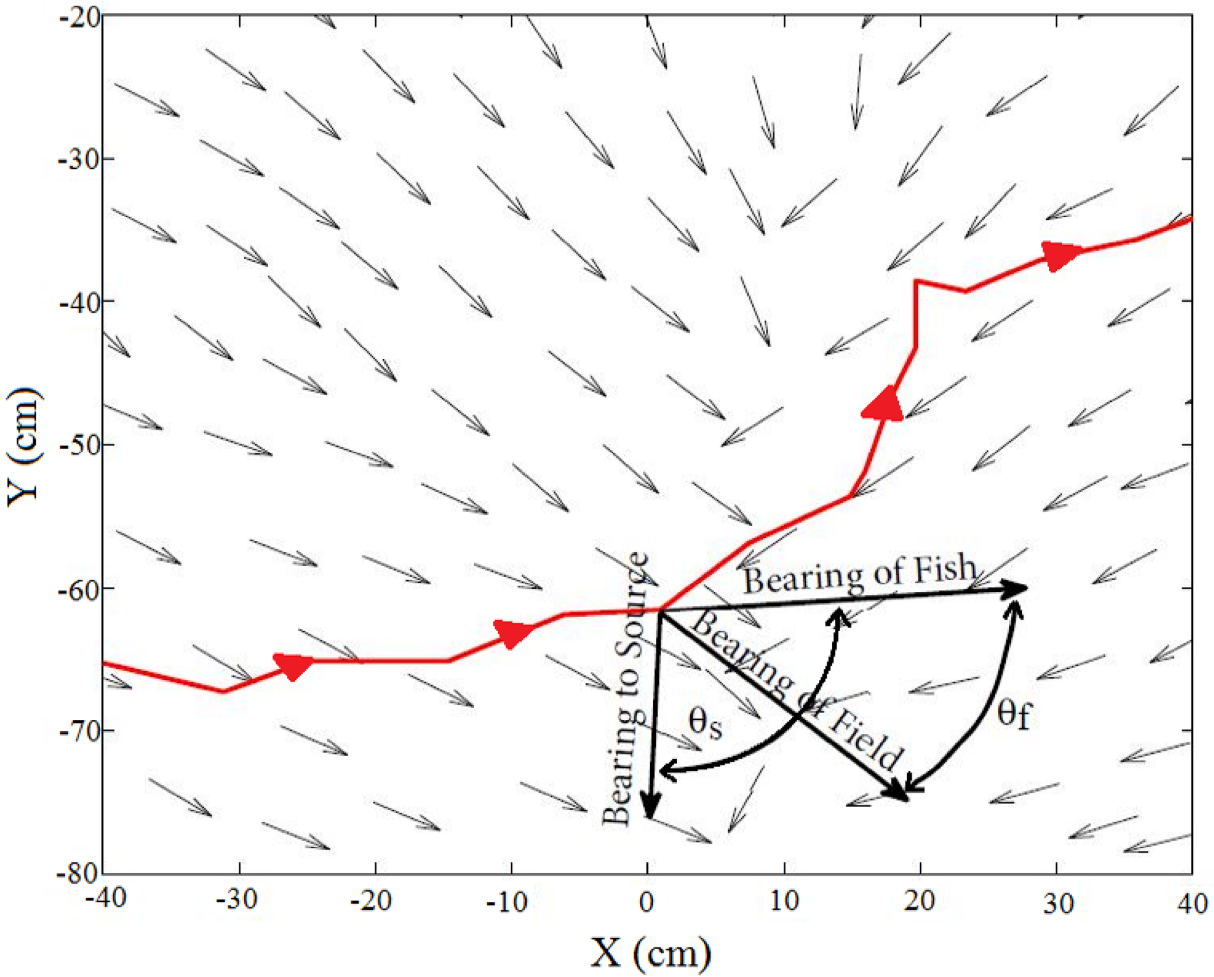
Difference angle of the fish’s bearing relative to the sound source (located at X = 0 cm), *θ*_*S*_, and the local particle acceleration vectors, *θ*_*F*_, at a given location along an individual swimming trajectory. Small arrows indicate local particle acceleration vectors (normalized for visual comparison), and the solid line indicates a sample swimming trajectory. Difference angles were calculated with reference to the origin (i.e. both difference angles in the example would have a negative value).

The second set of analyses examined the relationship between the orientation of individual fish to different components (sound pressure and particle motion) of the sound field and its source. Movement data from the full 150 s test period was used in this analysis (i.e., the first 30 s was not excluded). To accomplish this we calculated both the difference angle between the fish’s bearing relative to the sound source as well as the difference angle to the sensory field (particle acceleration vector) following Zeddies and others [5–9] (Fig 4). If sound pressure alone mediated phonotaxic responses, we hypothesized that fish would swim either directly away from the source (180°) or exhibit zig-zag movements to assess changes in relative sound pressure (as described for the mottled sculpin). Alternatively, if particle motion detection alone was the basis of orientation, the difference angle of the fish to the particle acceleration vectors was expected to be both nearly constant [47] and in line with the particle motion vector; in other words, it would be a relatively constant 0° [7–9].

Swimming trajectories were determined for each fish that swam within 30-cm of an active speaker at a 3 Hz sampling frequency. The position of each fish was evaluated 5 s before and after coming within 30-cm of the speaker, so fish were monitored to distances that might occasionally exceed 30-cm (some up to 125 cm). The entire treatment period was evaluated for each fish found in this space. The *x* and *y* coordinates of each fish’s head were used to determine both their distance from the source and orientation relative to measured sound fields. To test whether they orientated differently as they approached and then left the sound field, difference angles were analyzed separately as fish swam towards and away from the speaker. Both the difference angle relative to the speaker,*θ*_*S*_, and difference angle relative to the local particle acceleration vector,*θ*_*F*_, were calculated from the fishes trajectory in the *xy*-plane (Fig 4). Contributions of particle acceleration in the z-direction were ignored because particle acceleration magnitudes were similar throughout the enclosure in all three-directions (S4 Fig) and fish movement was laterally restricted. When fish were not located at a specific measurement point, the vector was interpolated linearly. Finally, difference angles were binned at 10-cm increments from the source and circular statistics used to calculate the mean angle, standard deviation, and vector strength [48]. Vector strength was used as a measure of the directional tendency of fish to move in a specific direction relative to either the source or particle acceleration axes (i.e. a value of 0 indicates difference angles were uniformly distributed, while values close to 1 indicates a concentration in one direction). The Rayleigh test was used on each group of binned difference angles to test whether they differed from random (P<0.05) [48]. Bearing to the speaker was used to compare swimming trajectories of fish when the sound was off (control) and then while it was on (treatment). This type of comparison could not be made using particle motion as no sound was played during controls. Sound pressure level at the fish’s location was also calculated along each individual swimming track and binned with the mean value and standard deviation calculated to determine if sound pressure might act as a threshold for behavior change.

## Results

### Fish distribution and avoidance

**Fig 5:**
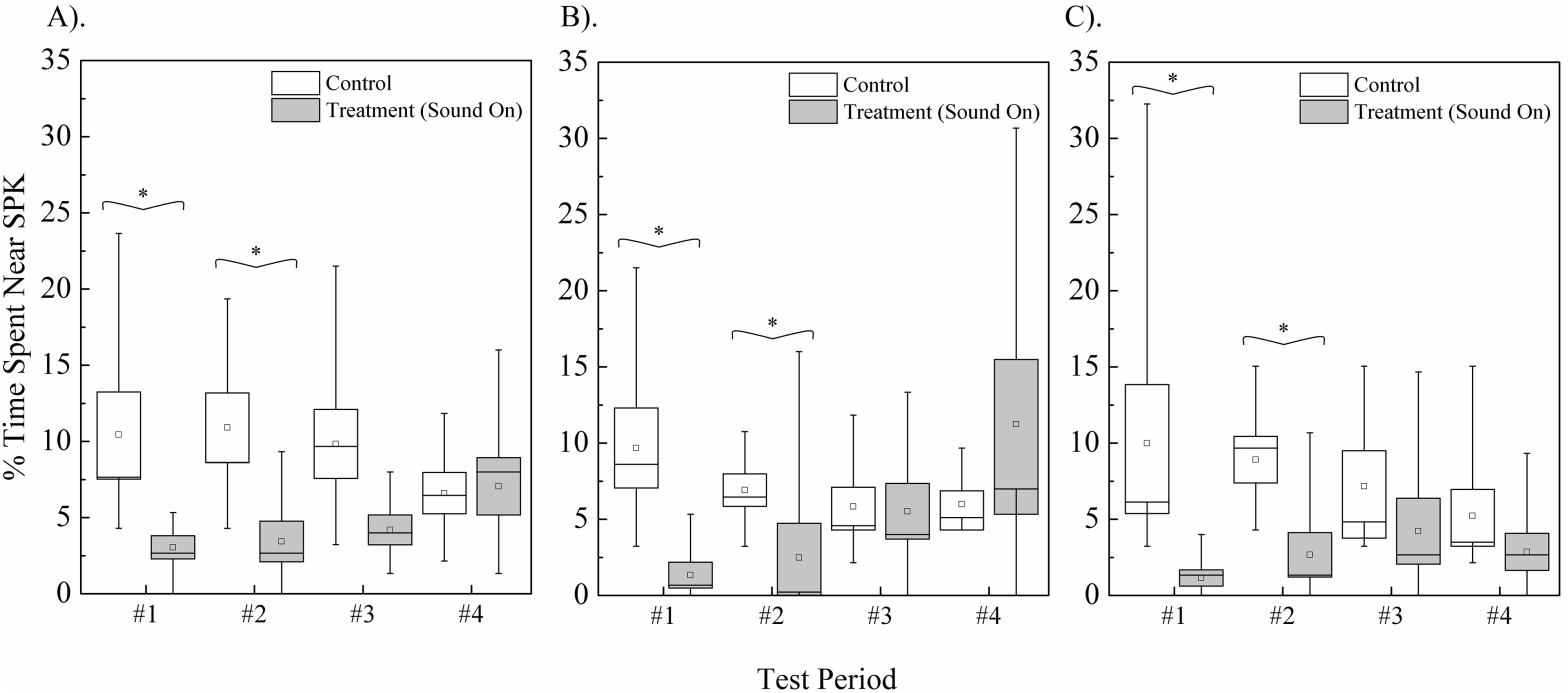
Percent time (A) common carp, (B) silver carp, and (C) bighead carp spent within 30-cm of a speaker before and during activation. Box plots illustrate data quartiles, mean (line), median (squares), and whiskers represent minimum and maximum values. Data from each species was analyzed separately with Mann-Whitney pair-wise comparisons, with (*) denoting mean times with significant difference P<0.05.

The median percent time fish spent within 30-cm of speakers during all 4 control periods (which a separate analysis showed not to differ between trials) was 9.5% [4.3, 11.8] (median [1^st^ and 3^rd^ quartiles]) for common carp, 7.0% [4.3, 9.7] for silver carp, and 7.9% [3.2, 11.8] for bighead carp. During the first playback the median percent time fish spent within 30-cm of the active speaker decreased to 3.0% [1.3, 5.3] for common carp, 1.3% [0.0, 2.3] for silver carp, and 1.1% [0.0, 1.3] for bighead carp, a decrease of at least two-thirds of control values (Fig 5). A similar decrease was observed for all three species during the second playback. No measurable change was observed for any species during either the third or fourth trials (P>0.05).

### Orientation

**Fig 6:**
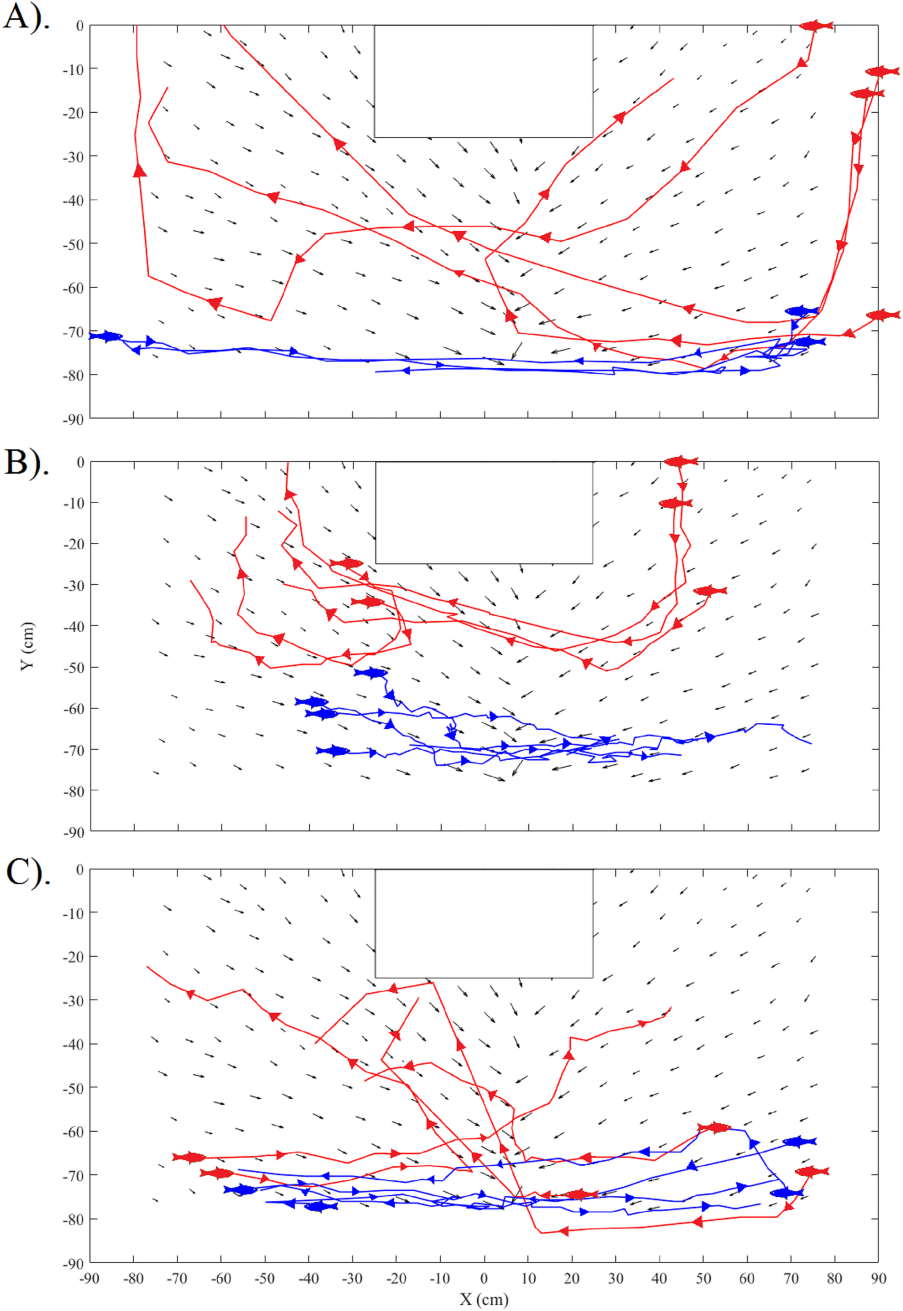
Representative responses of (A) common carp, (B) silver carp, and (C) bighead carp during control (blue lines) and treatment (red lines). Particle acceleration vectors are shown for reference. A fish symbol denotes the start and arrows indicate direction of movement. Note trajectories follow a curvilinear path parallel to local particle acceleration vectors during treatment periods, while trajectories follow paths parallel to the enclosure wall during control periods.

**Fig 7:**
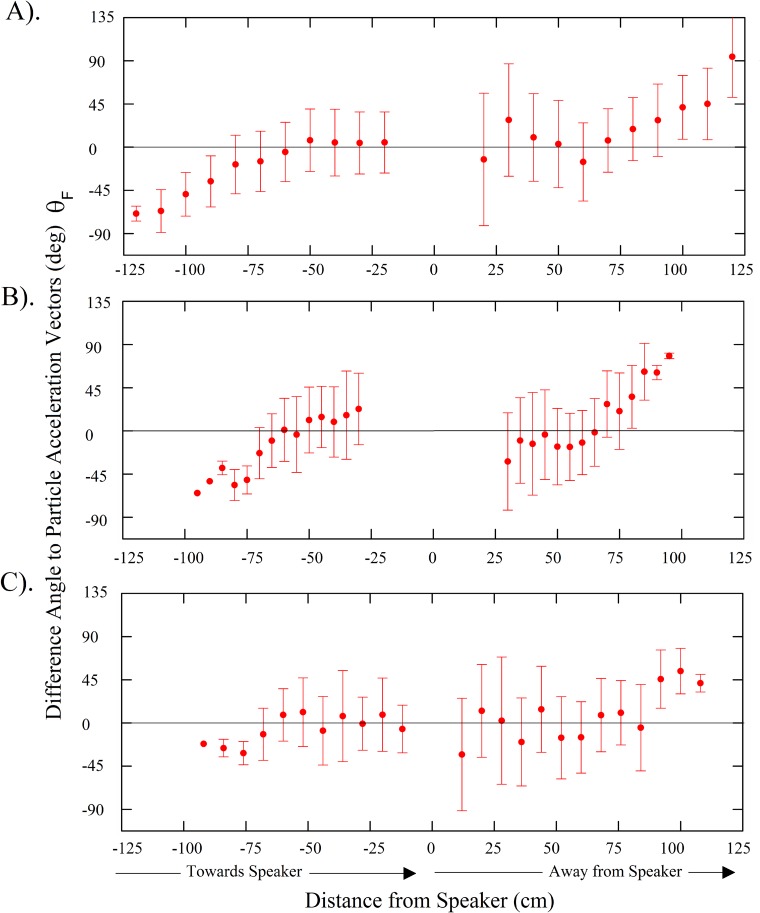
Difference angles of fish bearing relative to the local particle acceleration vectors for (A) common carp, (B) silver carp, and (C) bighead carp. Difference angles were calculated along swimming trajectories of fish that swam within 30-cm of an active speaker during playback. Trajectories were analyzed from all four trials. Negative distances indicate movement towards the speaker, while positive indicate movement away. Bars are the standard deviation.

**Fig 8:**
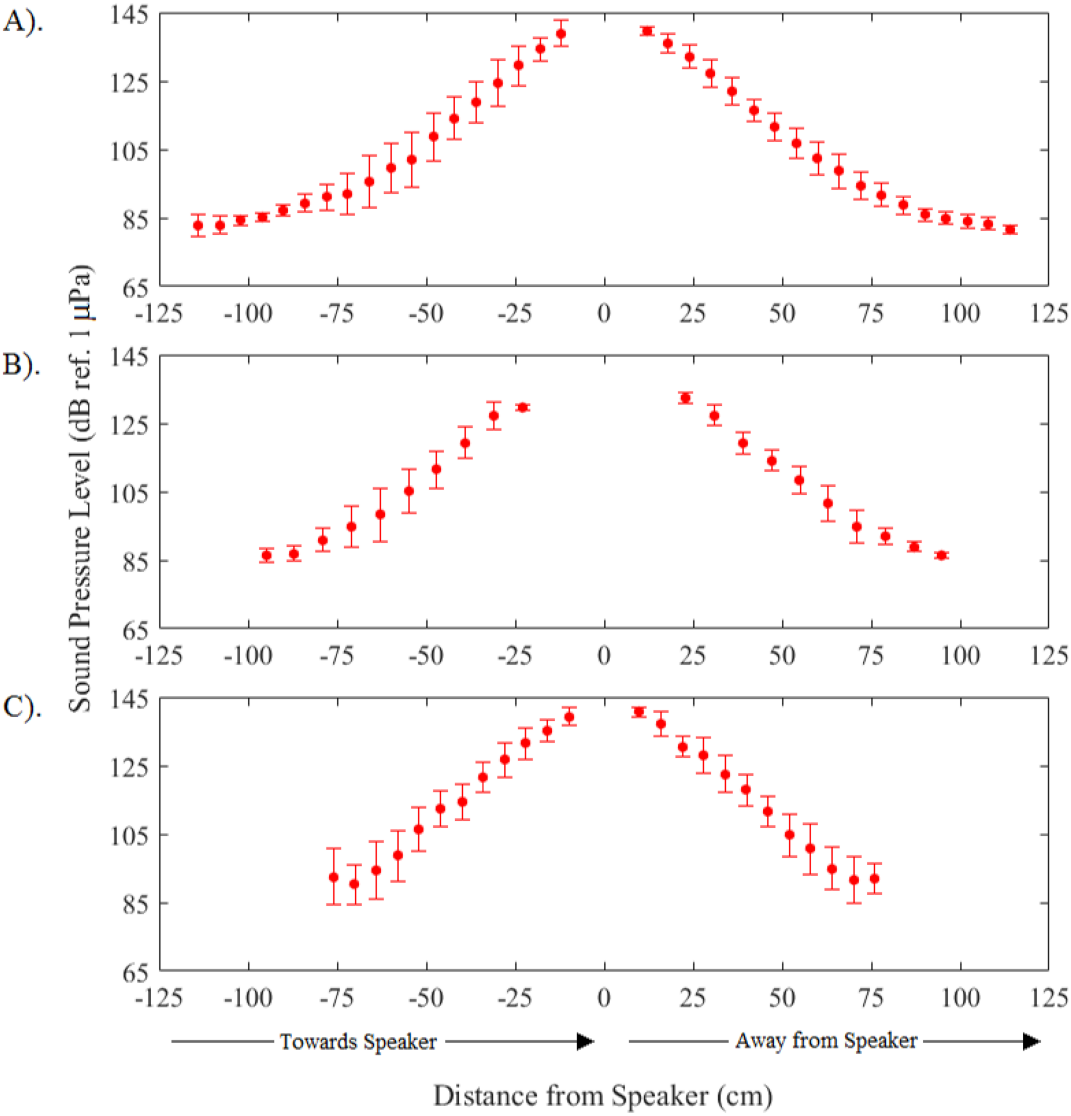
Mean sound pressure level along swimming trajectory of (A) common carp, (B) silver carp, and (C) bighead carp. Trajectories were analyzed from all four trials. Negative distances indicate movement towards the speaker, while positive indicate movement away. Bars are the standard deviation.

**Fig 9:**
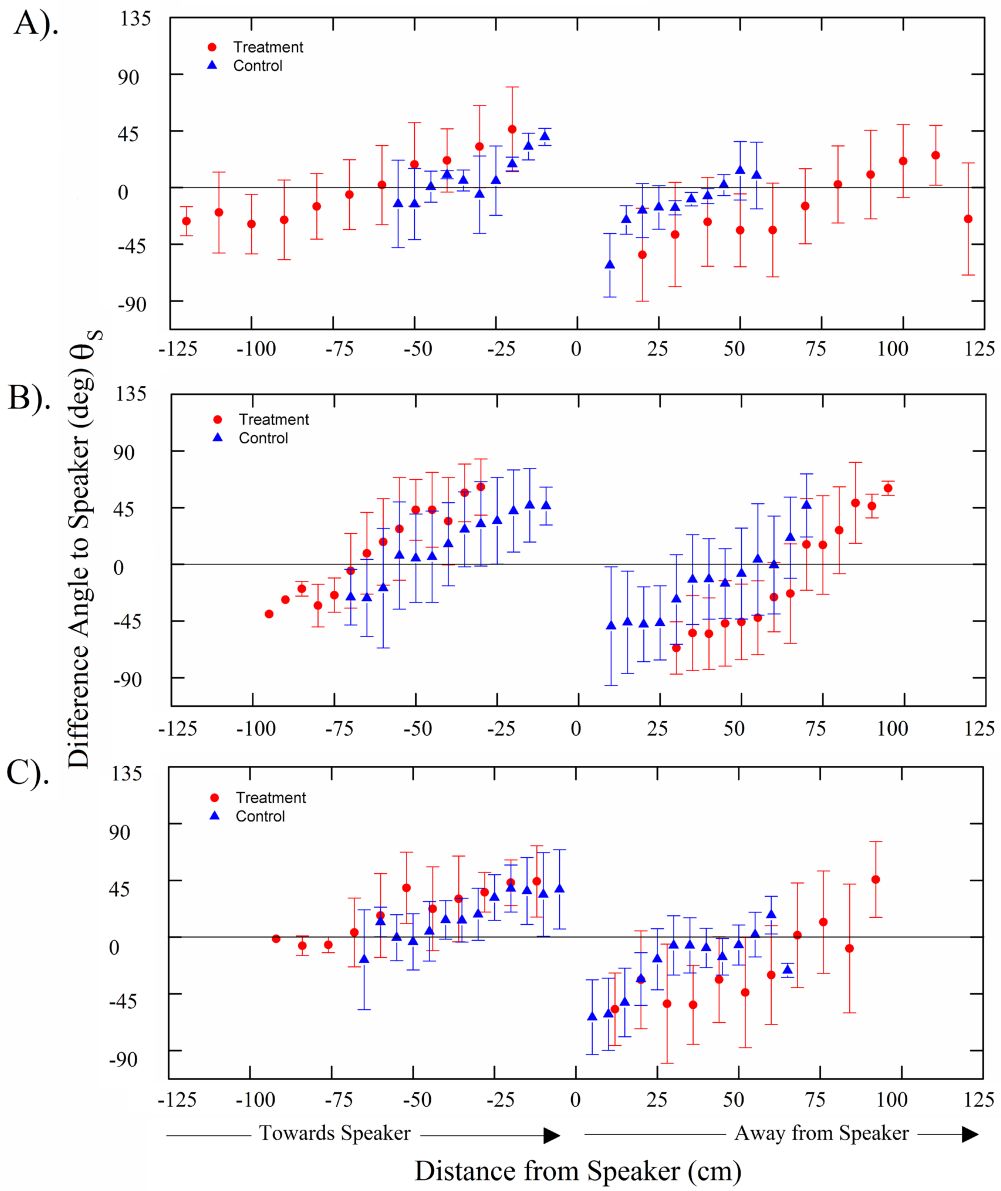
Difference angles of fish bearing relative to the speaker for (A) common carp, (B) silver carp, and (C) bighead carp. Difference angles were calculated along swimming trajectories of fish that swam within 30-cm of an active speaker during playback (treatment ●). Difference angles relative to the speaker are provided for 8 trajectories during control periods (control ▲). Trajectories were analyzed from all playbacks treatments. Negative distances indicate movement towards the speaker, while positive indicate movement away. Bars are the standard deviation.

A total of 45 common carp, 29 silver carp, and 26 bighead carp swimming trajectories were tracked and analyzed throughout all four trials. Plots during control periods showed that carps tended to follow the boundary walls at a distance of about 10–40 cm (Fig 6). In contrast, when the sound was turned on, individual fish showed a strong tendency to slowly turn while gradually increasing their swimming speed, thus resulting in their avoiding the location of the active speaker as they swam along curved tracks about 20–30 cm from the active speaker (Fig 6). Notably, the fish consistently maintained a nearly 0° orientation to local particle acceleration vectors while pursuing this behavior (Fig 7). Analyses showed that their exposure to sound pressure along the swimming trajectories consistently increased during approach and declined during avoidance at similar rates (Fig 8). No changes in swim paths, or apparent zig-zagging behavior were noted as fish approached or swam away from the sound source and a few fish seemed to employ c-starts. While common carp and bighead carp did not enter areas of the arena where sound pressure exceeded 140 dB (ref. 1 μPa), silver carp stayed further away and did not enter areas where sound pressure exceeded 130 dB (ref. 1 μPa). The difference angle to the sound source for all three carps showed similar trends with no apparent difference in mean angle when the sound was off (control) or on (treatment) (Fig 9). In both cases, difference angles started slightly negative and then increased as the fish approached the sound source, reaching 45° when within 30-cm, and followed a similar relationship as fish swam past the speaker. Further, error bars also did not increase dramatically near the source suggesting that carp swam in a very consistent, oriented fashion.

When the sound was on, all fish exhibited a high degree of directional tendency with respect to particle acceleration vectors (vector strength > 0.7 at distances between 30–120 cm for common carp, 30–95 cm for silver carp, and 30–108 cm for bighead carp). The difference angle to particle acceleration vectors varied when fish started to swim away from the speaker (i.e. vector strengths at bin locations within 30-cm of the speaker were below 0.7 for all species). Visual inspection of the plots suggested this variation was seemingly caused by fish reversing direction and moving out of alignment with the particle acceleration vectors for brief periods of time. Difference angles to the particle acceleration vectors differed from random for all three species up to a distance 80 cm from active speakers (Raleigh, P<0.05).

## Discussion

This study found that silver, bighead, and common carp exhibited negative phonotaxis when exposed to the sound of a complex, broadband sound in a dark, featureless environment but that this response habituated. Avoidance behaviors were strongly and consistently characterized by individual fish swimming along a curvilinear trajectory when sound pressure reached about 130–140 dB (ref. 1 μPa) (at a distance of 30 cm) from a hidden speaker and then swimming parallel to the axes of local particle acceleration before leaving the sound field. All carp followed extremely consistent trajectories with a nearly perfect 0° orientation to the axes of local particle acceleration. All three carp species showed very similar behaviors. Given the comparable hearing abilities of these species, it is not surprising that their avoidance responses and orientation strategies were similar. These results suggest that while pressure sensitive fishes such as carp and other ostariophysians may become aware of aversive sound by detecting changes in sound pressure, they likely then use particle motion to orient avoidance responses in the absence of visual landmarks. Presumably this would be the case in turbid river waters.

This study appears to be the first to describe the acoustic basis of negative phonotaxis in any fish and shows a clear role for particle motion, at least when visual landmarks are not available. Although carp are sensitive to sound pressure, and do appear to sense it as indicated by their reluctance to enter fields greater than 140 dB (ref. 1 μPa), all three carp species appeared to primarily use particle motion to orient away from the sound source as indicated by their near perfect orientation to the axes of particle acceleration. All three species of carp also started to orient to the particle acceleration axes when 50–75 cm from the sound source, a distance where sound pressure levels were marginally different from background. This set of observations strongly suggest that a particle acceleration of -25 dB (ref. 1 cms^-2^) is sufficient for these fish to orient and determine direction. The highly consistent nature of this behavior is consistent with both the directional nature of particle motion and how it is used by plainfin midshipman [7,8]. Notably, it differs notably from the zig-zagging behavior exhibited by the mottled scuplin which employed sound pressure via their mechanosensory lateral line to locate sound sources [5,6]. Orientating to local particle motion provides an efficient (i.e. less time and energy expense) pathway away from the source in the absence of other cues. While this study does not isolate the role of particle motion detection at the inner ear from the lateral line, ablation of the plainfin midshipman fish lateral line did not reduce phonotaxic behaviors [9], suggesting the role of input from the lateral line may be minimal in acoustically mediated orientation. The curved nature of the swimming paths observed in this study are consistent with avoidance behaviors of bigheaded carps and common carp previously seen to low frequency (< 5000 Hz) sound sources produced by air curtains in darkness [31–33]. The plainfin midshipman also swims in a directed linear fashion when approaching the sound produced by calling mates [7–9]. Similarly, the allis shad (*Alosa alosa*) is also known to swim at an angle of 180±30° to avoid sounds simulating those made by toothed whales (40 kHz clicks) [12–14] although its swimming paths have not been described as we have done.

While clearly showing that carp orient to particle motion, our results are consistent with the possibility that sound pressure may also play a role in initial phases of acoustically mediated behaviors of these species. Individuals started to avoid the active speaker when sound pressure levels reached 130–140 dB (ref. 1 μPa). When carp were near an active speaker, fish selected trajectories with sound pressure levels that increased and decreased gradually with minimal fluctuations. This differed markedly from the zig-zag trajectory used by mottled sculpin to sample the sound field and use for sound localization [5,6]. The role of pressure detection in source localization cannot be ascertained from these results, as this relationship is yet to be thoroughly defined for any species [9]. Taken together, our results suggest that while sound pressure may initiate the avoidance responses, particle motion ultimately guides carp movement in the absence of other cues.

Importantly, our study found that avoidance behavior in carp habituated with repeated testing, at least in darkness. This is not surprising, because habitation is common to all sensory cues used by organisms [49], and especially for continuous signals [50,51]. Transient hearing damage may have been one potential cause, but the exposure time of 150 s and sound pressure level 130–150 dB (ref 1 μPa) used in our study was far less than the 10 min period and 170 dB (ref 1 μPa) reported to cause a temporary threshold shift in goldfish (*Carassius auratus*), arguing against this. Our findings were undoubtedly influenced by the small size of our tank which provided no acoustic refuges and an anomalous sound field [52], especially because air surrounding the fiberglass tank creates a pressure release (i.e., sound pressure is zero but particle motion is not zero) along all boundaries which causes significant reflections of sound. This is different from natural water bodies that would only have a pressure release boundary at the water surface. Small tanks and shallow water also impact propagation of low frequency sounds due to boundaries interacting with sound wavelengths larger than the minimum dimensions of the tank or water depth [53]. To minimize issues related to small tank acoustics [54] we kept fish in the center of the tank and away from the complex sound field near the tank walls, sound field measurements were made at sufficiently fine spacing to capture any rapid changes in space, and both the sound pressure and particle acceleration fields were characterized. Although our findings that carps orient to particle motion cannot be directly applied to how fish might respond to natural sound fields in large open arenas, our basic finding that they respond to these fields is relevant.

While our findings are consistent with those of Vetter et al. [10,11] who found that groups of silver and bighead carp exhibited negative phonotaxis to a complex sound in a well-lit environment, they differ because we observed habituation and did not find that carp swam directly away from the sound source as they did. These apparent discrepancies can seemingly be explained by fundamental differences in testing protocols. Vetter et al. [10,11] used a well-lit arena where the speakers were easily visible and activated the sound as fish approached them, possibly facilitating a learning response to visual cues associated with sound (the speakers). Carp have excellent visual acuity and are likely capable of quickly learning visual cues when they are associated with sound. Notably, Vetter et al. [10,11] also did not measure fish tracks but rather changes in apparent lateral position of the centroid of entire groups and thus were unable to compare movements to sound pressure level or particle motion. Explicit tests of how fishes use sound with and without visual cues appear warranted.

This study also provides new information on acoustic behavior of the common carp, which is highly invasive in shallow water ecosystems. Common carp were similarly, albeit less responsive to complex sound than bighead and silver carp, as has been noted previously [55]. All three species exhibited a similar tendency to move parallel to the particle acceleration vectors out to a distance of 60 cm from the speaker. Zielinski et al. [33] also found consistent avoidance responses of all three species to a bubble curtain (a low frequency sound source). Although common carp are not reasonable surrogate for all aspects of bighead and silver carp invasions, they could be a conservative model for bigheaded carp (which are more difficult to capture and study) when testing acoustic deterrents because it is less sensitive and more readily available in areas not yet invaded by bighead or silver carp (i.e., headwaters of the Mississippi River).

The findings of this study strongly support the possibility that acoustic deterrents could be useful to help control silver, bighead, and common carp movement in rivers including the Mississippi River which is extremely turbid [56]. While earlier studies show general movement away from a sound source [10,11,37,38], our study clearly shows that particle motion is used for orientation and could be used to direct all species of carp away from an area. Nevertheless, carp barrier design should consider the fact that particle motion attenuates rapidly. One way to use this new understanding of the role of particle motion in darkness may be to design new types of acoustic deterrents to divert (vs. block) carp to swim along alternative paths (i.e. via an air curtain deflection screen; [33,57]). Acoustic deterrents have been effectively paired with bubble curtains to manipulate the distribution of sound, creating a sharp sound pressure gradient [34–36,57]. Nestler et al. [58] also proposed using directional transducers to create well defined sound fields to obstruct fish passage into water intake structures. Another possibility might be to employ lights and / or visual landmarks to provide additional information for orientation but this may not always be possible in river waters which often have poor clarity. New sounds and associated sets of stimuli warrant systematic study to see if improvement on the paradigm we tested might be possible.

## Conclusions

Behavioral responses of silver, bighead, and common carp to a stationary complex sound were observed to characterize whether and how these species avoid sound in the absence of visual cues. Plotting showed all three species exhibit an oriented avoidance response which habituated after two trials. Swimming trajectories correlated strongly with the axes of local particle motion by trending towards 0°. Fish also turned away from the speaker at a distance of 20–30 cm where the sound pressure level was above 140 dB (ref. 1 μPa). Future studies should examine how carp accomplish this type of orientation, how common it might be, and whether and how other sensory cues might enhance orientation capability. The findings of this study nevertheless suggest that acoustic deterrents could be used to control invasive carp, but that field testing is needed to address issues including range, the roles of other sensory stimuli in different environments, habituation, and non-target effects, especially in low light environments. Different sounds might also be considered.

## Supporting information

S1 Fig The power spectra of (A) the sound pressure measures in the tank and (B) signal played during experiments. The sound pressure level was measured 5 cm in front of the speaker.
(TIF)

S2 Fig Plan view of sound pressure level in dB (ref 1 μPa) at 2000 Hz in the entire enclosure at a depth of 15 cm from the tank bottom. The speaker is hidden behind a plastic screen and located at 0 cm on the X-axis, with the center of the projector face 15 cm from the tank bottom.
(TIF)

S3 Fig Sound pressure level in dB (ref 1 μPa) at 2000 Hz as a function of radial distance from the source. Measurements plotted along radius at π/4, π/2, and 3π/4. The box in the center of enclosure causes the break in measurements along π/2 radius. Theoretical attenuation (dashed line) for shallow water is calculated using Eq. 12–13 from Akamatsu et al. [53].
(TIF)

S4 Fig Particle acceleration in X-, Y-, and Z- direction in dB (ref 1 cms^-2^) as a function of radial distance from the source along the enclosure centerline. Box in the center of enclosure causes the break in measurements.
(TIF)

S5 Fig Time-series plot of mean distance between (A) common carp, (B) silver carp, and (C) bighead carp and active speaker during first playback. Open gray circles denote raw positions while black squares are the mean distance with standard error bars. The x axis shows sound starting from o when the sounds was turned on. Note that fish maintained a relative constant distance from the speaker after 30 seconds.
(TIF)
