# Supplementary figures and images for: Silver, bighead, and common carp orient to acoustic particle motion when avoiding a complex sound
Source: PLoS One. 2017 Jun 27;12(6):e0180110. doi: 10.1371/journal.pone.0180110 (PMC5487063; doi:10.1371/journal.pone.0180110)

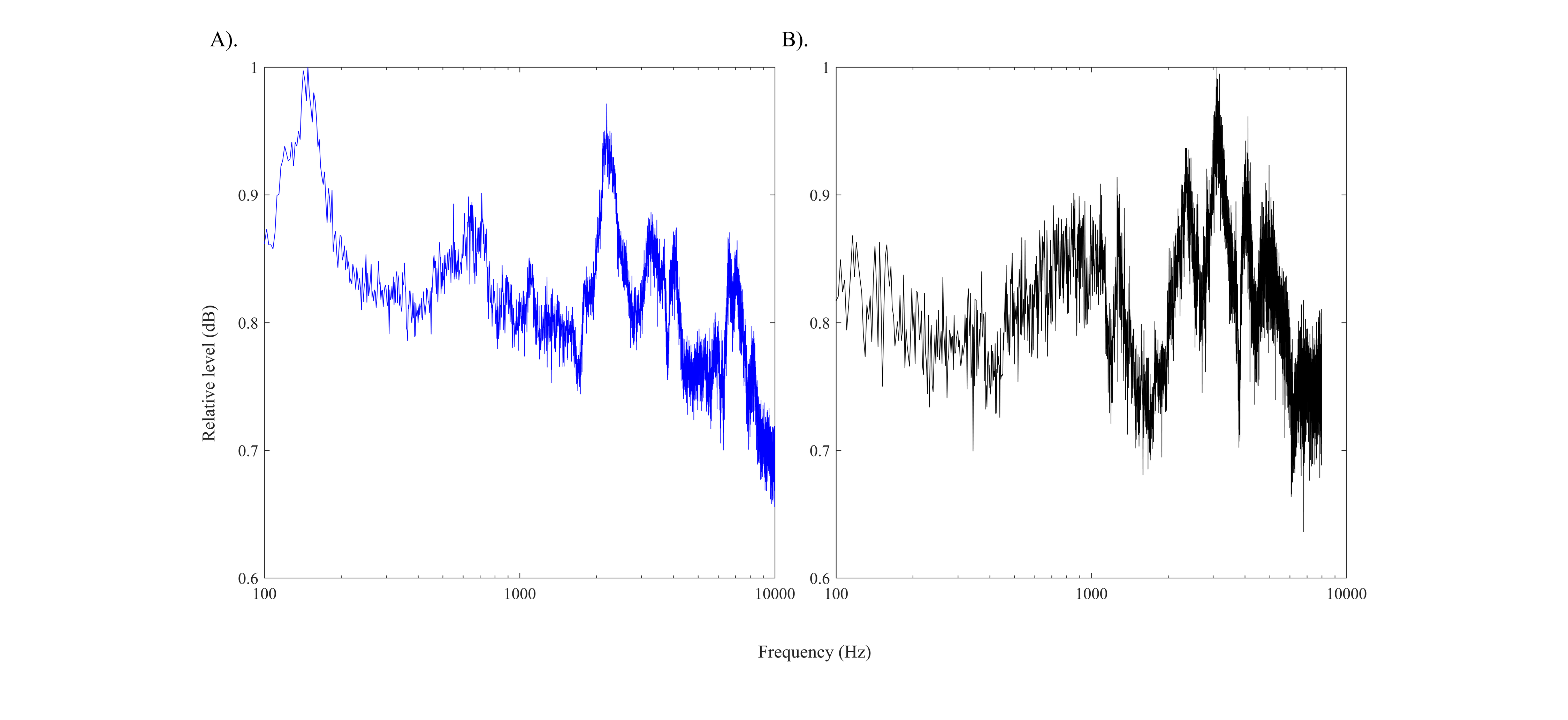

Supplement: S1 Fig — The sound pressure level was measured 5 cm in front of the speaker. (TIF) [file pone.0180110.s001.tif]

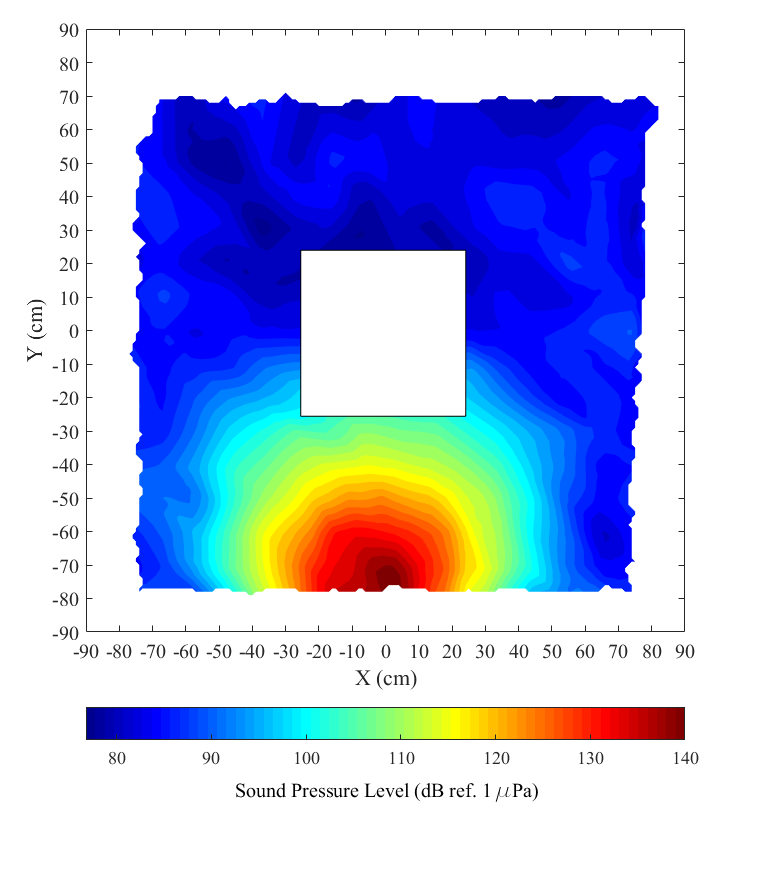

Supplement: S2 Fig — The speaker is hidden behind a plastic screen and located at 0 cm on the X-axis, with the center of the projector face 15 cm from the tank bottom. (TIF) [file pone.0180110.s002.tif]

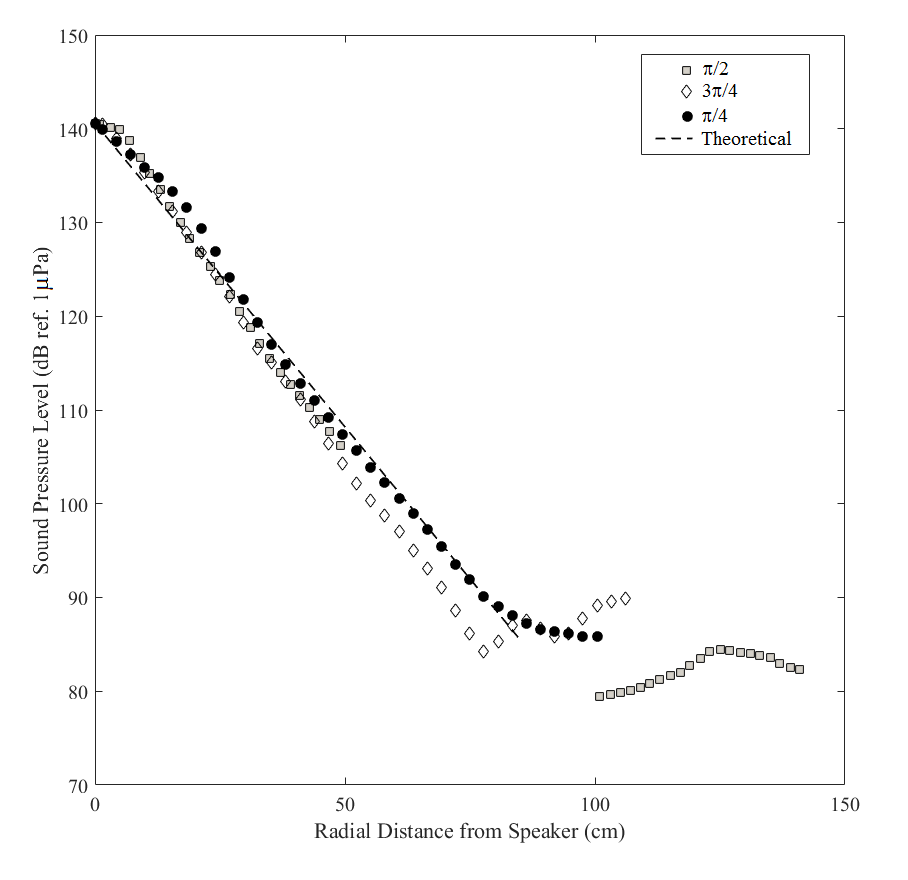

Supplement: S3 Fig — Measurements plotted along radius at π/4, π/2, and 3π/4. The box in the center of enclosure causes the break in measurements along π/2 radius. Theoretical attenuation (dashed line) for shallow water is calculated using Eq. 12–13 from Akamatsu et al. [53]. (TIF) [file pone.0180110.s003.tif]

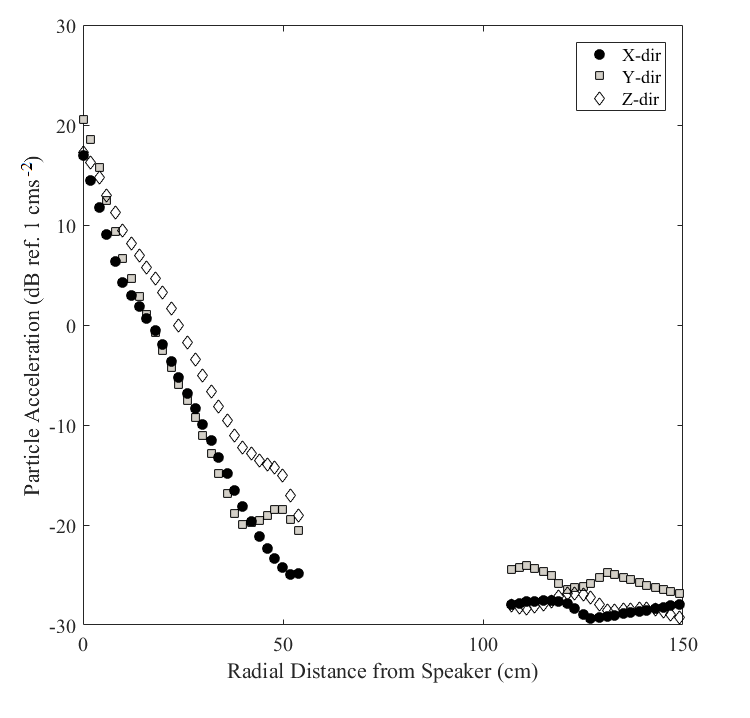

Supplement: S4 Fig — Box in the center of enclosure causes the break in measurements. (TIF) [file pone.0180110.s004.tif]

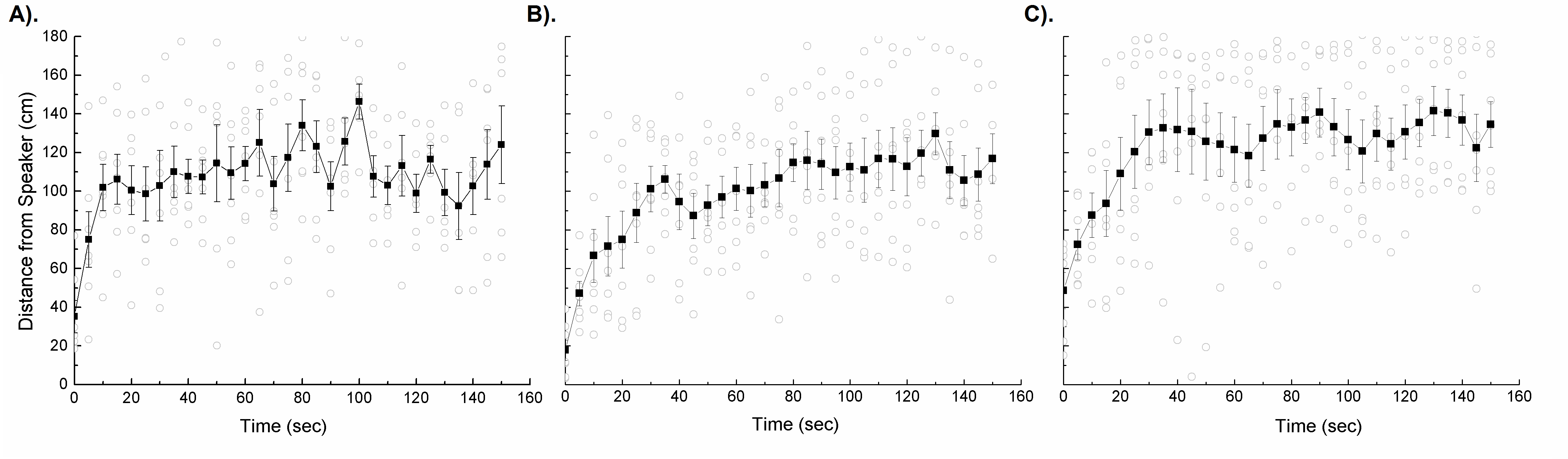

Supplement: S5 Fig — Open gray circles denote raw positions while black squares are the mean distance with standard error bars. The x axis shows sound starting from o when the sounds was turned on. Note that fish maintained a relative constant distance from the speaker after 30 seconds. (TIF) [file pone.0180110.s005.tif]
